# Characterization of a broad-based mosquito yeast interfering RNA larvicide with a conserved target site in mosquito *semaphorin-1a* genes

**DOI:** 10.1186/s13071-019-3504-x

**Published:** 2019-05-22

**Authors:** Keshava Mysore, Ping Li, Chien-Wei Wang, Limb K. Hapairai, Nicholas D. Scheel, Jacob S. Realey, Longhua Sun, David W. Severson, Na Wei, Molly Duman-Scheel

**Affiliations:** 1 Department of Medical and Molecular Genetics, Indiana University School of Medicine, South Bend, IN USA; 2 Eck Institute for Global Health, The University of Notre Dame, Notre Dame, IN USA; 3 Department of Biological Sciences, The University of Notre Dame, Notre Dame, IN USA; 4 Department of Civil and Environmental Engineering and Earth Sciences, The University of Notre Dame, Notre Dame, IN USA; 5 Department of Life Sciences, The University of the West Indies, St. Augustine, Trinidad, Trinidad and Tobago

**Keywords:** *Aedes aegypti*, *Anopheles gambiae*, *Culex quinquefasciatus*, *Saccharomyces cerevisiae*, Arbovirus, Dengue, Malaria, Insecticide, RNA interference, Nervous system

## Abstract

**Background:**

RNA interference (RNAi), which has facilitated functional characterization of mosquito neural development genes such as the axon guidance regulator *semaphorin-1a (sema1a)*, could one day be applied as a new means of vector control. *Saccharomyces cerevisiae* (baker’s yeast) may represent an effective interfering RNA expression system that could be used directly for delivery of RNA pesticides to mosquito larvae. Here we describe characterization of a yeast larvicide developed through bioengineering of *S. cerevisiae* to express a short hairpin RNA (shRNA) targeting a conserved site in mosquito *sema1a* genes.

**Results:**

Experiments conducted on *Aedes aegypti* larvae demonstrated that the yeast larvicide effectively silences *sema1a* expression, generates severe neural defects, and induces high levels of larval mortality in laboratory, simulated-field, and semi-field experiments. The larvicide was also found to induce high levels of *Aedes albopictus*, *Anopheles gambiae* and *Culex quinquefasciatus* mortality.

**Conclusions:**

The results of these studies indicate that use of yeast interfering RNA larvicides targeting mosquito *sema1a* genes may represent a new biorational tool for mosquito control.

**Electronic supplementary material:**

The online version of this article (10.1186/s13071-019-3504-x) contains supplementary material, which is available to authorized users.

## Background

The use of larvicides, chemical or microbial agents that kill mosquito larvae in the aquatic habitats in which they breed, is a key component of integrated mosquito control programmes. *Aedes* mosquitoes, the primary vectors of dengue, yellow fever, chikungunya and Zika viruses, lay eggs in water-holding containers located in urban areas [1], and these containers are readily treated with larvicides. *Culex* mosquitoes, the principle vectors of West Nile virus and lymphatic filariasis [2, 3], are also susceptible to larvicides, which are used to treat the catch basins in which the mosquitoes often breed [4]. When used in addition to insecticide-treated nets (ITNs) and indoor residual spraying (IRS), larviciding is cost-effective for control of *Anopheles* (malaria vector) mosquitoes in urban settings in which breeding sites are few, fixed and findable [5]. Due to increases in insecticide resistance to existing pesticides and heightened concerns for unintended adverse effects of pesticides on non-target organisms, new larvicides to combat existing and emerging mosquito-borne illnesses must be identified. For example, *Bacillus thuringiensis israelensis* (Bti), methoprene briquettes, and granular formulations of temephos are often used to treat *Aedes* breeding sites [6–9], but resistance to these pesticides is emerging [10–16]. Furthermore, methoprene can be toxic to invertebrate non-target species [7], and regulatory approval for temephos was not renewed in the USA in 2015 [17]. Pyrethroid resistance in *Culex pipiens* complex mosquitoes is a global problem, and high levels of resistance have been observed in larvae collected in the field (reviewed in [18]). High levels of Bti resistance have also been observed in *C. pipiens* larvae [19]. Furthermore, although ITNs and IRS have been the backbone for malaria vector control, resistance to insecticides used in these applications is an increasing problem [20], and neither intervention can combat *Anopheles* mosquitoes that bite or rest outdoors [20].

Ingested double-stranded RNA (dsRNA) molecules may represent a new class of species-specific insecticides [21]. RNA interference (RNAi) has been applied for functional characterization of mosquito neurodevelopment genes, including the analysis of *Ae. aegypti sema1a* gene function in embryos and larvae [22–24]. Microinjection of custom synthetically synthesized small interfering RNAs (siRNAs) targeting the *Ae. aegypti sema1a* gene demonstrated that embryonic silencing of this gene results in multiple nerve cord phenotypes in embryos [22]. Delivery of siRNA to *Ae. aegypti* larvae using chitosan nanoparticles demonstrated that Sema1a also plays key roles in the developing larval nervous system [24]. Loss of *sema1a* function in larvae results in defective olfactory receptor neuron targeting, as well as projection neuron defects coincident with a collapse in the shape and structure of the antennal lobe and individual glomeruli [24]. Larval silencing of *sema1a* also results in optic lobe defects, including visual sensory neuron targeting defects and improper formation of the retinotopic map [23]. In addition to pursuing functional characterization of neurodevelopmental genes such as *sema1a*, we recently began to examine the potential for translating RNAi technology to the field for mosquito control. To this end, we have pursued large-scale screens for siRNAs that target genes required for *Ae. aegypti* [25] and *An. gambiae* [26] larval survival. A subset of the siRNAs/genes screened, including siRNA larvicide 460, the subject of our present investigation, were assessed due to the conservation of the target sequences for these siRNAs in multiple vector mosquito species. Given the larvicidal activity of siRNA 460 and the conservation of its target site in mosquito *sema1a* genes (see below), a gene that we had coincidentally studied in previous investigations [23, 24], we decided to pursue detailed characterization of this larvicide. Due to the high degree of conservation of the target site (a site that had not been targeted in our previous studies [22–24]), it was hypothesized that interfering RNA corresponding to this site could function as a broad-range mosquito larvicide.

The application of RNAi for mosquito control requires cost-effective means of RNA production and an effective RNA delivery system. Although chitosan/siRNA effectively silences the *Ae. aegypti sema1a* gene [23, 24], the current inability to mass produce interfering RNAs in a cost-effective manner presently hinders the advancement of this technology as a means of controlling mosquitoes in the field. In an effort to develop an economical means of producing RNAi larvicides, as well as a mechanism for field delivery of these larvicides to mosquitoes, we have begun to engineer *Saccharomyces cerevisiae* (baker’s yeast) strains that express short hairpin RNA (shRNA) corresponding to larval lethal genes identified in the siRNA screens [25, 26]. Use of yeast, which is readily consumed by mosquito larvae, facilitates cost-effective synthesis of interfering RNA, which is produced through yeast cultivation [27]. Our initial studies focused on characterization of yeast strains that produce shRNAs which selectively targeted either *Ae. aegypti* [25, 26] or *An. gambiae* [25, 26] mosquito larvae. In this study, *S. cerevisiae* was engineered to express shRNA corresponding to the conserved siRNA 460 mosquito *sema1a* target site, a site that is not known to be conserved in non-target organisms. The results of this investigation indicate that this yeast larvicide functions as a broader-based mosquito insecticide that kills *Ae. aegypti*, *Ae. albopictus*, *Cx. quinquefasciatus* and *An. gambiae* larvae, but that does not impact non-target arthropods. This larvicide may represent a new intervention for the biorational control of multiple species of disease vector mosquitoes.

## Methods

### Animal rearing

*Aedes aegypti* Liverpool-IB12 (LVP-IB12) strain mosquitoes, *Ae. albopictus* Gainesville strain (from the NIH Biodefense and Emerging Infections Research Resources Repository, NIAID, NIH (BEI Resources) strain MRA-804, Manassas, VA), *Cx. quinquefasciatus* JHB strain (BEI Resources strain NR-43025) and *An. gambiae* G3 strain (BEI Resources strain MRA-112) mosquitoes were used in this investigation. Mosquitoes were maintained generally as described previously [28], except that sheep blood (purchased from HemoStat Laboratories, Dixon, CA, USA) was delivered to adult females through a Hemotek artificial membrane feeding system (Hemotek Limited, Blackburn, UK). The mosquito strains were kept in an insectary maintained at 26 °C, at ~80% relative humidity, and under a 12 h light:12 h dark cycle with 1 h crepuscular periods at the beginning and end of each light cycle.

### Larval soaking experiments

siRNA #460 was discovered in an siRNA screen for mosquito larval lethal genes that was conducted as described [25, 26]. As noted above, a subset of the siRNAs screened corresponded to target sites that were found to be conserved in mosquitoes using an algorithm developed by Scott Emrich (University of Tennesee), which will be described elsewhere. Sequences/genes identified using the algorithm were screened if they corresponded to genes that are orthologs of *D. melanogaster* larval lethal genes and are expressed throughout *Ae. aegypti* larval development as discussed previously [25, 26]. For the screen, custom siRNAs purchased from Sigma-Aldrich (St. Louis, MO, USA) corresponded to the following target sequences: #460: 5′-AUU AUC GUC GCG GUG ACG GAU-3′ in *sema1a* (*AAEL019771*; formerly *AAEL002653*) and a control sequence which is not present in any of the mosquito species: 5′-GAA GAG CAC UGA UAG AUG UUA GCG U-3′ [29]. As previously discussed [25, 26], the larval soaking screens were performed in duplicate using the general methodology described by Singh et al. [30] on 20 first-instar larvae soaked in 0.5 µg/µl siRNA for four hours. Following these treatments, the larvae were reared and evaluated as described in the World Health Organization (WHO) [31] larvicide testing guidelines, and screen data were assessed through use of the Fisher’s exact test. All error reported in this study corresponds to standard error of the mean (SEM).

### Generation of yeast larvicide strains, yeast culturing, and tablet preparation

Both stable and transiently transformed yeast strains corresponding to the control siRNA sequence were generated previously [25, 26] and used in the present investigation (control strain sequence information is included in Additional file 1). The methodology used to generate these control strains [25, 26] was employed for generation of plasmid-based transient and stably transformed sema.460 strains. A DNA oligonucleotide cassette encoding sema.460 shRNA (see sequence in Additional file 1) was purchased from Invitrogen Life Technologies (Carlsbad, CA, USA). For generation of the plasmid-based strain, the #460 shRNA expression cassette (which was designed to include *Bam*HI and *Xho*I compatible sticky ends to facilitate cloning) was cloned into the multiple cloning site in *pRS426 GPD* [32], a non-integrating yeast shuttle vector marked with *URA3.* The cassette was cloned into the *Bam*H1 and *Xho*I sites, which placed it downstream of the strong and constitutively active *GPD* promoter and upstream of the *cyc1* terminator in *pRS426 GPD* [32]. Following confirmation of the clone through restriction digestion (with *Bam*H1 and *Kpn*I*)* and sequencing (Additional file 1), the resulting plasmid was used for transformation of *S. cerevisiae* strain *BY4742* (genotype *MATα his3Δ1 leu2Δ0 lys2Δ0 ura3Δ0*, [33]). Yeast transformants were screened through selection on SC minimal media lacking uracil. A more detailed description of these methods can be found in Mysore et al. [34].

For generation of stable sema.460 transformants, DNA encoding sema.460 shRNA and the *cyc1* terminator was ligated downstream of *Gal1*, a strong inducible promoter [35], to generate the sema.460 expression cassette (Additional file 1), which was subsequently inserted into the multiple cloning sites of two yeast integrating plasmid shuttle vectors, *pRS404* and *pRS406* [36] (sequences for these vectors, which bear *TRP1* and *URA3* selection markers, respectively, are available through Addgene [37, 38]). To generate the *pRS406* clone, the sema.460 shRNA-encoding DNA and *cyc1* terminator fragment were excised with *Bam*H1 and *Kpn*I digestion and gel purification from the pRS426 GPD clone described above. This fragment was ligated to the *Gal1* promoter fragment, which was custom-synthesized by Integrated DNA Technologies (Coralville, Iowa, USA) and included 5′ *Xho*I and 3′ *Bam*HI compatible sticky ends. This *Gal1* promoter fragment was ligated upstream of the *sema.460-cyc1* terminator fragment, which retained a 5′ *Bam*H1 compatible sticky end. The resulting expression cassette, which had 5′ *Xho*I and 3′ *Kpn*I compatible ends, was inserted at the *Xho*I and *Kpn*I sites in the multiple cloning site of *pRS406* [36]. To generate the *pRS404* [36] clone, the *Gal1-sema.460-cyc1* terminator expression cassette was extracted from *pRS406 via* restriction digestion with *Not*I and *Kpn*I and gel purification, then ligated into these sites in *pRS404* [36]. The two *pRS406-sema.460* and *pRS404-sema.460* plasmids, which were verified *via* restriction digestion and sequencing, facilitated integration of two copies of the sema.460 shRNA expression cassette at both the *trp1* and *ura3* loci in the *CEN.PK S. cerevisiae* strain (genotype *MAT*a/α *ura3-52/ura3-52 trp1-289/trp1-289 leu2-3_112/leu2-3_112 his3* Δ*1/his3* Δ*1 MAL2-8C/MAL2-8C SUC2/SUC2*, [39]). Selection of doubly transformed *S. cerevisiae* was performed using synthetic complete media lacking tryptophan and uracil. PCR and sequencing were utilized to further verify integration of the transgene (sequences are provided in Additional file 1) at both the *trp1* and *ura3* loci.

Following yeast strain generation, control [25] and #460 shRNA expression strains were grown through shake culture as discussed previously [25]. In summary, the yeast strains were grown under standard conditions in synthetic media to an OD_600_ of 3.0. For galactose induction of the stable transformant strains, yeasts were cultured in 20 ml SCD medium containing 20 g/l glucose until early stationary growth phase, after which time the cells were harvested by centrifugation, transferred into 200 ml of fresh SCD medium containing 20 g/l galactose and 2 g/l glucose, then cultured at 30 °C and 250× *rpm* for 18 h (OD600 ~ 3.0). Dried inactivated yeast tablets were then prepared as described [34]. In summary, for preparation of each tablet, 40 ml of liquid culture was transferred to 50 ml conical tubes and pelleted by centrifugation. The pellet was placed in a 70 °C water bath for 5 min to heat-kill the yeast. The yeast pellet was then transferred to a 2 ml tube and centrifuged briefly. After removing any remaining media from the supernatant, the tubes were left open in an incubator or a food dehydrator at 30 °C for 48 h to evaporate remaining media. The resulting 70 mg tablet was either used immediately or stored for up to one week in the capped 2 ml tube at −20 °C.

### Conduction of larvicide assays in mosquitoes

#### Laboratory trials

Laboratory larvicide trials were performed in the insectary as described [26]; these assays conformed to WHO [31] guidelines, and a more detailed description of these procedures is included in [34]. Larvae that consumed heat-inactivated control or shRNA #460 interfering RNA larvicide tablets were evaluated in parallel in multiple biological replicate experiments (see figure legends for details), with each biological replicate experiment consisting of at least three replicate containers per condition. For each container replicate assay, one control or experimental tablet was placed in 500 ml plastic cups containing 50 ml of distilled water and 20 newly hatched first-instar larvae. When larvae reached L4, 300 µl of 6% w/v liver powder (MP Biomedicals, Solon, OH, USA) was provided as a nutritional supplement. Mosquito survival was monitored daily until all control-treated animals had emerged as adults. For statistical analysis of larval mortality assays, the percentages of mortality from larvicide- *vs* control-treated containers were combined from replicate experiments and transformed to arcsine values for evaluation with a t-test. Dose-response curves were generated as detailed previously [25] using data combined from three biological replicate experiments, with each biological replicate experiment including four replicate containers per condition (one yeast tablet fed to 20 larvae in 50 ml of water as discussed above). For the tablets used in these assays, yeast expressing control interfering RNA was mixed with sema.460 yeast to various concentrations to generate the dried inactivated yeast tablets. Data from all replicate experiments were pooled for analysis. LD_50_ values with 95% confidence intervals were calculated, as discussed previously [25] and recommended by the WHO [31] larvicide testing guidelines, through generation of a log dosage-probit mortality regression line using regression analysis in SPSS software. The mortality in the control group, which was less than 2%, was corrected according to Abottʼs formula [40] as recommended [31]. Linear regression analysis was performed separately using Microsoft Excel software.

#### Assessment of F1 progeny of survivors

200 L1 larvae were treated with 10 sema.460 or control tablets, which were placed in 1 liter of water. Following treatment, adult survivors (F0) were bred, and eggs (F1) collected from gravid females were hatched. For each of two biological replicates of the above experiment, three biological replicate containers, each with 200 larvae, were treated with 10 sema.460 or control tablets. The percentages of mortality were combined from replicate experiments and transformed to arcsine values for evaluation with ANOVA followed by Tukey’s pairwise comparison.

#### Individual rearing/feeding experiments

Individual larvae were subjected to larvicide treatment using a slightly modified version of the methodology described above. First, 4 mg mini yeast pellets were prepared by pelleting 5 ml of stably transformed sema.460 or control yeast cultured as described above. Pellets were then processed as detailed above, except that drying times were reduced to one day. Individual larvae were then placed in a container holding 50 ml distilled water, a mini yeast pellet, and 50 µl of 6% w/v liver powder. For each of three replicate experiments, 20 individual sema.460-treated and 20 individual control-treated larvae were assessed. Data from replicate experiments were combined and assessed through Chi-square analysis.

#### Simulated field trials

Experiments were performed and analyzed generally as described above, but with two modifications. In one set of experiments, larvicide trials were conducted using 100 ml of rainwater (instead of sterile distilled water) that had been collected in South Bend, IN, USA, during summer 2018. Another set of experiments involved the use of larvae that had been hatched from the F2 generation of an *Ae. aegypti* strain recently generated from eggs collected in ovitraps in Trinidad, Trinidad and Tobago during summer 2018.

#### Semi-field trials

Semi-field larvicide trials were performed in an outdoor rooftop laboratory in Notre Dame, IN during July and August 2018. These assays, which were conducted using *Ae. aegypti* LVP-IB12 strain mosquitoes, conformed to the WHO [31] larvicide testing guidelines. The trials were performed in a SansBug 1-Person Free-Standing Pop-Up Mosquito-Net tent (Hakuna Matata Tents, Ontario, Canada). The mesh in the tents (472 openings/cm) prevented the exit of mosquitoes from the test site, as well as the entry of macrobiota into the tent while the assays were being conducted. The 30-l containers (depth = 46 cm) used for these assays were filled with 26 l of water, 20 larvae, and a single yeast tablet (prepared from control or larvicidal stably-transformed yeast according to the methodology used in reference [34] and described above) and were covered with mesh to provide a second level of containment. Three biological replicate experiments, each with three replicate containers per condition, were completed. During the testing period, temperatures ranged from 13.5 °C to 42.0 °C. The mean daytime temperature during this trial period was 27 °C, and the mean nighttime temperature was 23 °C.

#### Whole mount in situ hybridization and immunohistochemistry

The *Aae sema1a* riboprobe was synthesized according to the Patel [41] protocol and utilized to perform *in situ* hybridization experiments on fourth-instar *Ae. aegypti* brains using methodology previously described [24, 42]. L4 larvae were fixed for these *in situ* experiments, which were performed in triplicate, just prior to the time that treated animals would typically die. Processed brains were mounted and imaged using a Zeiss Axioimager equipped with a Spot Flex camera. Following imaging, mean gray values (average signal intensity over the selected area) were calculated for quantification of digoxigenin-labeled transcript signals in control *versus* experimental brains using FIJI ImageJ software. A t-test was used to statistically analyze transcript quantification data combined from the three experiments.

Immunohistochemical staining experiments were performed in triplicate using previously described methodology [43, 44]; mAb nc82 anti-Bruchpilot [45] (Developmental Studies Hybridoma Bank, University of Iowa, Iowa City, Product nc82, which was deposited by E. Buchner) and TO-PRO-3 iodide (Molecular Probes, Eugene, OR) were used in these experiments. Larvae from four replicate containers per condition were fixed, processed, and evaluated in each of three biological replicate experiments. Processed brains were mounted and then imaged through use of a Zeiss 710 confocal microscope and Zen software, and these images were evaluated through use of FIJI ImageJ and Adobe Photoshop CC 2018 software. For quantification of antibody staining intensity, mean gray values were calculated as discussed previously [46]. In summary, for assessment of mean gray values, the average signal intensity for digoxigenin-labeled transcript signal in control or larvicide-treated brains using Adobe Photoshop CC 2018 software. Data combined from the three replicate experiments were statistically analyzed using a t-test.

### Toxicity assays in non-target species

#### Drosophila melanogaster

The survival of *D. melanogaster* larvae (from the *w*^*118*^ stock [47] obtained from the Bloomington *Drosophila* Stock Center, Bloomington, IN, USA) that had fed on sema.460 or control interfering RNA yeast was assessed using methodology derived from Murphy et al. [48]. These studies were performed at 22 °C under ambient laboratory illumination (12 h light: 12 h dark). In these assays, one pellet of dried inactivated sema.460 or control interfering RNA yeast that had been prepared as described above was resuspended in 300 µl of water and mixed with standard fly food medium at a ratio of 1:1 by weight. In each of two biological replicate assays, the yeast-food mixture was fed to 10 larvae in a 1.5 ml Eppendorf tube secured with a cotton stopper. After four days, a moistened lab wipe was added to the tube to increase humidity and provide a surface for pupation. Following pupation, tubes were opened and moved to a vial in which adult emergence was assessed. The number of adults that emerged from each tube was recorded as a measure of survival. Data from two biological replicate experiments were combined and assessed using the Fisher’s exact test.

#### Daphnia spp.

*Daphnia pulex* and *Daphnia magna* were acquired from Carolina Biologicals (Burlington, NC, USA). Toxicity was assessed using 10 adults of each species in each of three biological replicate assays. These studies were conducted at 22 °C under ambient laboratory illumination (12 h light: 12 h dark) in COMBO medium containing 0.0001% sodium selenium [49]. A single yeast pellet (sema.460 or control) was dissolved in 50 ml of distilled water, and 10 ml of the solution was fed to the animals daily for five days. Survival was assessed on a daily basis over a 10-day trial period. Survival data from three replicate experiments were combined and assessed with the Fisher’s exact test.

## Results

### *S. cerevisiae* expressing shRNA corresponding to the sema1a gene kill *Ae. aegypti* larvae

**Fig. 1:**
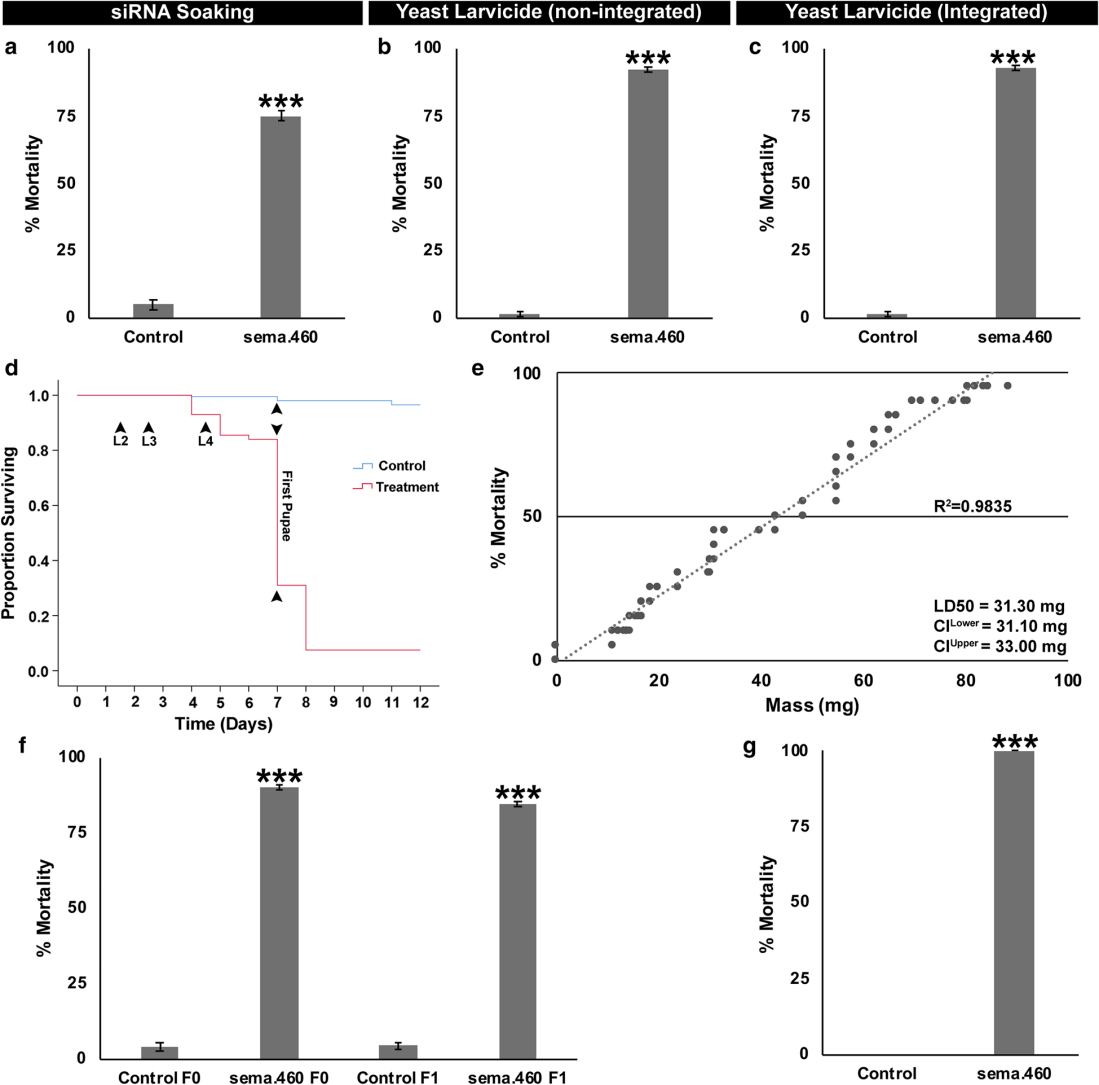
Silencing *Aae sema1a* induces larval mortality. **a** siRNA #460, which corresponds to *sema1a*, was identified in a screen [25] in which *Ae. aegypti* larvae soaked in 0.5 μg/μl siRNA #460 died (compare to control siRNA-treated larvae which survived). Screen data from two replicate experiments (*n* = 20 larvae/replicate) were combined and assessed with the Fisher’s exact test. shRNAs corresponding to the #460 or control siRNA sequences were expressed in *S. cerevisiae* from a plasmid (**b**) or following integration of two copies of the shRNA expression cassettes into the *S. cerevisiae* genome (**c**). Larval consumption of inactivated dried yeast interfering RNA tablets corresponding to the #460 target sequence induced significant *Ae. aegypti* larval mortality (**b**, **c**). Data shown in panels **b** (*n* = 12 replicate containers/treatment, each with 20 larvae for a total of 240 larvae/treatment) and **c** (*n* = 12 replicate containers/treatment, each with 20 larvae for a total of 240 larvae/treatment) were combined from three biological replicate experiments and analyzed with t-tests. For each container replicate in **b**, **c**, and all subsequent figures, one 70 mg yeast tablet was fed to 20 larvae unless indicated otherwise. **d** *Ae. aegypti* larval consumption of inactivated dried sema.460 tablets prepared from stably-transformed strains (which were used for the assays in this panel and all subsequent trial data reported in this investigation) induced death during the L4 larval or pupal stages (days 4–8), while larvae that consumed control yeast survived to adulthood. **e** A dose-response curve shows that larval mortality is linearly correlated (*R*^2^ = 0.9835) with the dose of sema.460 provided to larvae. Each point on the graph corresponds to the percentage mortality observed in a single container replicate assay performed on 20 larvae (data were compiled from three biological replicate experiments). sema.460 LD_50_ values with upper and lower confidence limits (CL) are shown. **f** Larvae that survived treatment with sema.460 were bred, and the susceptibility of their offspring to sema.460 was assessed. No significant differences in larvicide activity were noted in the F0 *vs* F1 generations (for each generation, *n* = 6 container replicates/condition, each with 200 larvae that were fed with 10 tablets); data were combined from two biological replicate experiments and analyzed by ANOVA. **g** When sema.460-treated larvae reared as individuals were fed with one 4 mg tablet, 100% mortality was observed (all control-treated individuals survived). Data on a total of 60 individuals/treatment (combined from three biological replicate experiments) were analyzed by Chi-square analysis. The data presented in panels **a**–**c**, **f** and **g** correspond to mean percentages of mortality, with error bars here and in all subsequent figures representing SEM. ***P < 0.001 with respect to control-treated larvae in all panels

siRNA #460, which corresponds to a conserved target sequence in mosquito *sema1a* genes (Additional file 2: Table S1), was uncovered in an *Ae. aegypti* siRNA soaking screen for larval lethal genes [25]. In the screen, siRNA #460 induced 75 ± 5% larval death (Fig. 1a, *χ*^2^ = 44.17, *df* = 1, *P* = 3.02E^−11^ *versus* control siRNA treatment) following brief soaking treatment of first instar *Ae. aegypti* larvae. Based on the results of the screens, a non-integrating multi-copy yeast shuttle plasmid in which shRNA corresponding to the #460 target sequence was placed under control of a constitutive promoter, was constructed and used to transform *S. cerevisiae.* Yeast strain #460, as well as a yeast strain that expresses a control shRNA with no known target site in mosquitoes [25], were used in the preparation of an inactivated dried yeast tablet [34]. Larvae which ingested control yeast tablets survived (Fig. 1b), but 92 ± 1% of larvae that fed on yeast interfering RNA #460 died (Fig. 1b, *t*_(13)_ = 2.17, *P* = 7.48E^−18^ *versus* control treatment).

Based on the high levels of mortality induced by yeast that had been transiently transformed (Fig. 1b), it was determined that it would be beneficial to generate yeast that are stably transformed with the shRNA #460 expression cassette. Following generation of a strain bearing two stably-integrated copies of the shRNA #460 expression cassette (hereafter referred to as sema.460), dried, heat-inactivated yeast larvicide sema.460 tablets were prepared from the strain. Yeast larvicide sema.460, as well as a comparable stable transformant strain expressing control shRNA (described in reference [25] and hereafter referred to as the control) were fed to *Ae. aegypti* larvae. Yeast larvicide sema.460 induced 93 ± 1% mortality in *Ae. aegypti* larvae when 20 larvae were reared and treated in a single container (Fig. 1c, *t*_(12)_ = 2.20, *P* = 1.04E^−17^ *versus* control yeast treatment). When larvicide activity was assessed in F1 progeny of sema.460-treated F0 survivors, no significant differences in the levels of larval mortality induced by sema.460 were observed in the F0 *versus* F1 generations (Fig. 1f, *F* = 688, *df* = 3, *P* = 2.45E^−20^, sema.460-F0 *vs* F1 Tukey: *P* = 0.07). In all of these sema.460 experiments (Fig. 1b, c), no dead larvae were observed in these containers. In contrast, when larvae were reared and treated as individuals, 100% of sema.460-treated larvae died (Fig. 1g, *χ*^2^ = 155.60, *df* = 1, *P* = 1.04E^−35^), and the larval remains could be observed in each container for several days. Larvae treated with sema.460 larvicide died during the fourth instar or as pupae (Fig. 1d). Larval death was linearly correlated to the dose of sema.460 fed to the larvae (Fig. 1e; control mortality = 1.08 ± 0.33%; LD_50_ = 31.3 mg).

### Larval consumption of sema.460 yeast larvicide results in *Aae sema1a* silencing and severe defects in the *Ae. aegypti* larval nervous system

**Fig. 2:**
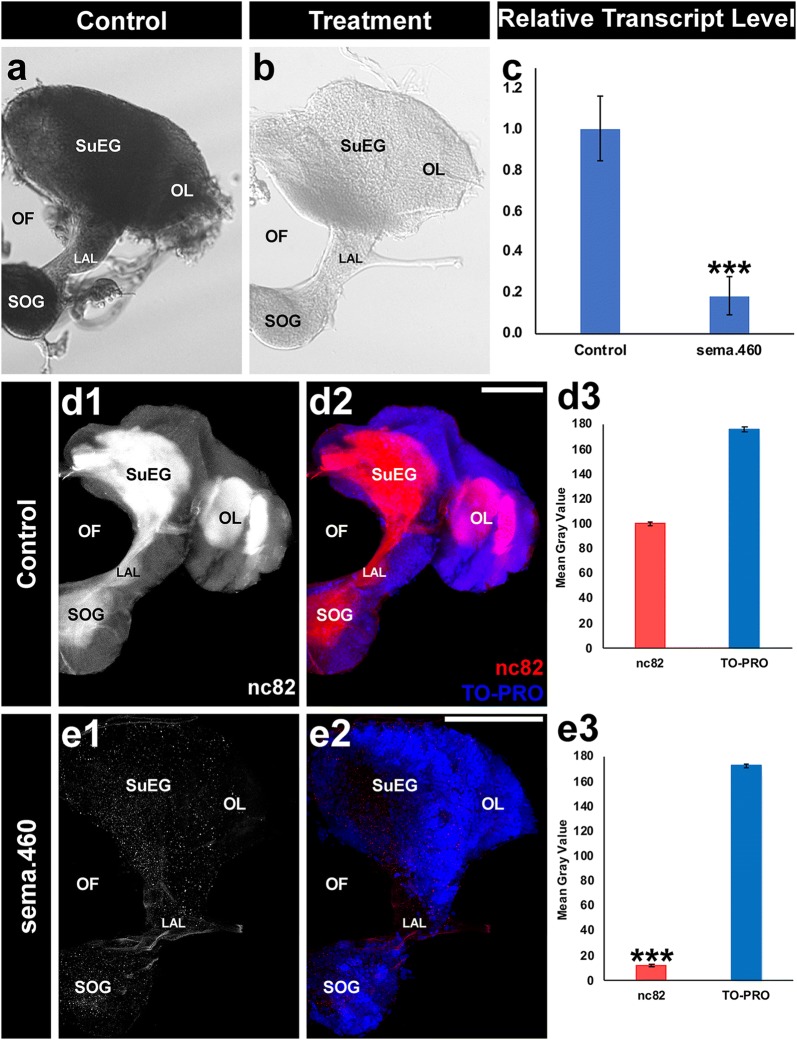
Yeast larvicide sema.460 effectively silences the *sema1a* target gene and induces severe neural defects in the *Ae. aegypti* L4 brain. Larval consumption of dried inactivated sema.460 tablets results in silencing of *sema1a* (**b**), which is normally expressed at high levels throughout the L4 larval brain ([24]; control-treated animal is shown in **a** for comparison). These results were quantified through analysis of mean gray value comparisons of sema.460- *versus* control-treated larvae (**c**; *n* = 53 sema.460-treated L4 brains; *n* = 57 control-treated L4 brains; data are represented as average mean gray values in **c**, as well as **d3** and **e3**). Average mean grey value analyses (compiled from three biological replicate experiments with 65 control-treated larvae in **d3** and *n* = 51 sema.460-treated larvae in **e3**) indicated that levels of nc82, which marks synaptic active zones (white in **d1**, **e1**; red in **d2**, **e2)**, were significantly reduced in the synaptic neuropil of L4 larvae, while TO-PRO nuclear staining (blue in **d2** and **e2**) levels were not significantly different. ***P < 0.001 with respect to control-fed larval brains (**c**, **e3**, analyzed by t-test). Representative L4 brains are oriented dorsal upward in this figure. *Abbreviations*: SOG, sub-esophageal ganglion; SuEG, supra-esophageal ganglion; LAL, larval antennal lobe; OF, olfactory foramen; OL, optic lobe

As previously reported [24], *Aae sema1a* transcripts are expressed broadly in the brain of L4 larvae (Fig. 2a). Quantification of *Aae sema1a* transcript levels in the L4 larval brain of animals fed with control (Fig. 2a) *versus* sema.460 (Fig. 2b) dried inactivated yeast larvicide tablets confirmed that larval consumption of sema.460 results in significant silencing of *sema1a* (Fig 2c; 82 ± 9% reduction of *sema1a* transcript was observed; *t*_(53)_ = 2.00, *P* = 1.57E^−57^). Based on previous studies [24], it was hypothesized that severe neural defects would be observed in *Ae. aegypti* larvae that had consumed yeast larvicide sema.460. Larval silencing of *sema1a* through chitosan/siRNA-mediated targeting of alternative sites in the *Aae sema1a* gene [24] resulted in significantly lower levels of nc82 antibody staining, which reveals expression of the active zone marker Bruchpilot (Brp; detected by nc82 antibody staining [45]). Similarly, Brp levels were significantly reduced (*t*_(43)_ = 2.01, *P* = 1.95E^−48^ *versus* control) in the L4 brains of larvae that had consumed sema.460 (Fig. 2e1–e3; 88 ± 9% reduction observed with respect to control-treated animals, which are shown in Fig. 2d1–d3). These severe neural defects correlated with the time of larval death observed in sema.460-treated larvae (Fig. 1d).

### Yeast larvicide sema.460 induces high levels of larval mortality in simulated-field and semi-field experiments

**Fig. 3:**
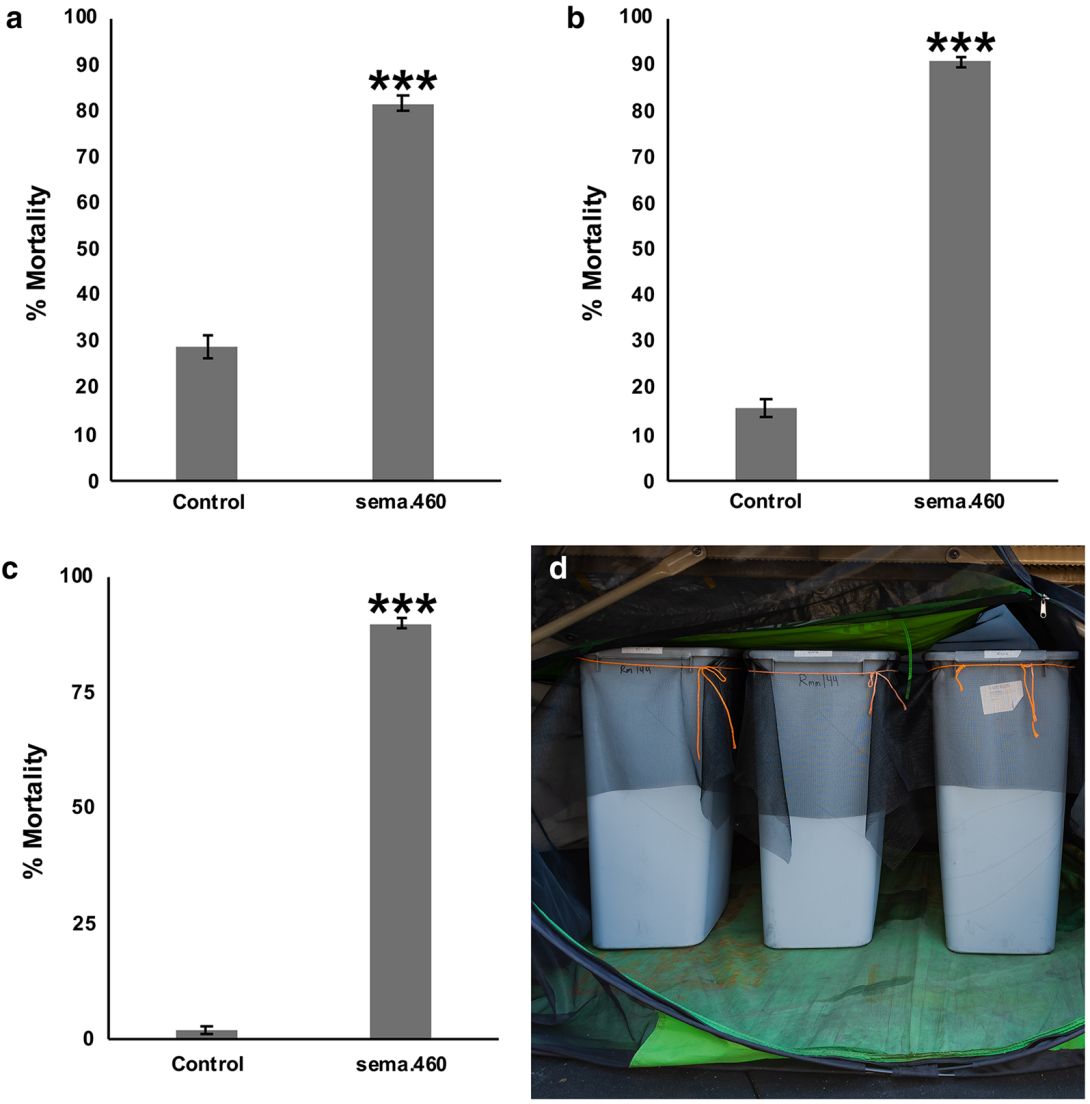
Simulated-field and semi-field evaluation of sema.460 activity in *Ae. aegypti.* **a** Activity of sema.460 was confirmed in trials conducted in rainwater. Data shown were compiled from two biological replicate experiments and represent results from six container assays, each with 20 larvae for a total of 120 larvae assessed/condition. **b** Activity was also confirmed in F2 larvae from a Trinidad field strain. Data were compiled from three replicate experiments conducted on a total of 240 larvae/condition that were assessed in 12 containers, each with 20 larvae. **c** The activity of sema.460 was also confirmed in semi-field trials. Data were compiled from three biological replicate experiments conducted on a total of 180 animals/treatment in nine replicate containers with 20 larvae each. **d** Semi-field trials were conducted in a contained outdoor roof top laboratory. Mean percentages of larval mortality are shown in **a**–**c**. Data in **a**–**c** were analyzed with t-tests; ***P < 0.001 when compared with control-fed larvae

The activity of sema.460 was next assessed under conditions that more closely simulate conditions encountered in the field. The activity of sema.460 was confirmed in insectary experiments that were conducted using rainwater rather than sterile distilled water (Fig. 3a), with 82 ± 2% larval mortality observed in sema.460-treated animals (*t*_(5)_ = 2.57, *P* = 9.13E^−05^ *versus* control-treated larvae). The activity of sema.460 was also assessed and confirmed in insectary experiments conducted on F2 larvae collected from a newly generated strain of *Ae. aegypti* mosquitoes established from eggs collected in Trinidad, Trinidad and Tobago (Fig. 3b). 91 ± 1% larval mortality was observed in sema.460-treated animals in these experiments (*t*_(11)_ = 2.20, *P* = 3.97E^−12^ *versus* control-treated larvae). Finally, sema.460 activity was assessed in semi-field experiments conducted in an outdoor roof top laboratory. Larger 30-l containers bearing 26 l of water, a size which better approximates common productive *Ae. aegypti* breeding sites found in the tropics [50], were used in these trials. Although little death was observed in containers in which larvae fed on control yeast tablets, 90 ± 1% of larvae in containers treated with sema.460 tablets died (Fig. 3c, d, *t*_(8)_ = 2.30, *P* = 6.87E^−12^ *versus* control-treated larvae).

### sema.460 functions as a broad-range mosquito larvicide

**Fig. 4:**
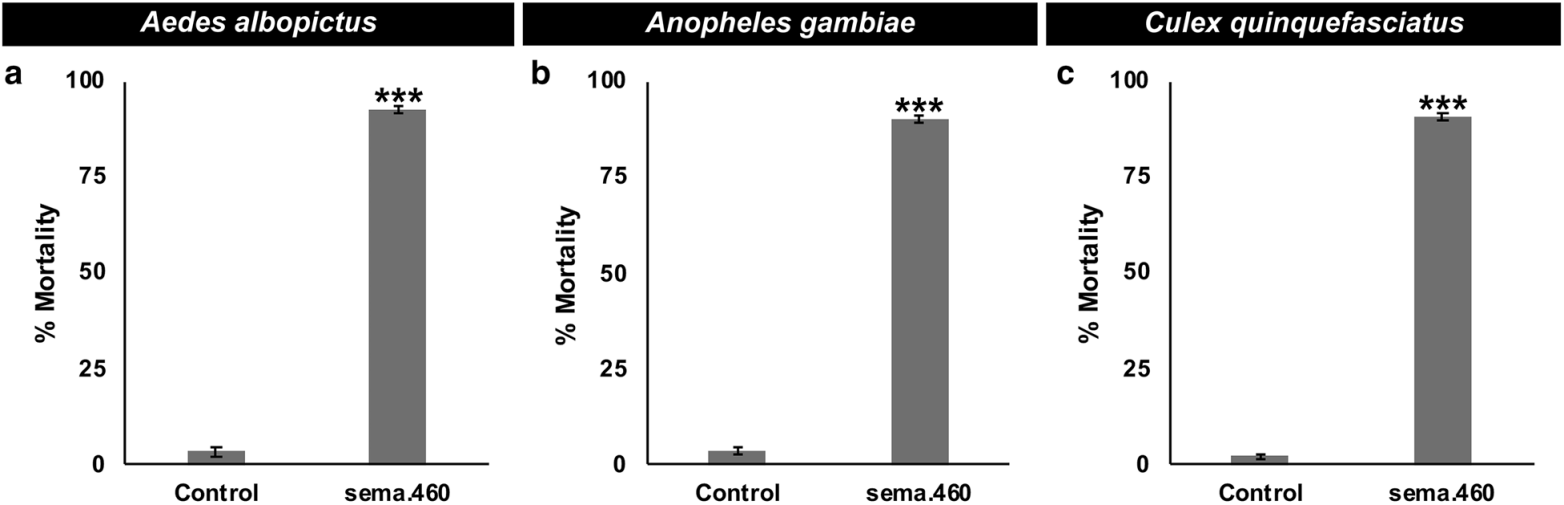
sema.460 yeast kills larvae of multiple mosquito species. Oral consumption of sema.460 results in high levels of *Ae. albopictus* (**a**), *An. gambiae* (**b**) and *Cx. quinquefasciatus* (**c**) larval mortality. **a** A total of 180 *Ae. albopictus* larvae were assessed in nine replicate containers/condition, each with 20 larvae. **b** 240 *An. gambiae* larvae were evaluated in 12 replicate containers/condition, each with 20 larvae. **c** 240 *Cx. quinquefasciatus* larvae were assessed in 12 replicate containers/condition with 20 larvae in each container. The results shown in this figure were compiled from three biological replicate experiments conducted on each mosquito species. Data shown represent mean larval mortalities. ***P < 0.001 in comparison to control-fed larvae (t-test)

**Fig. 5:**
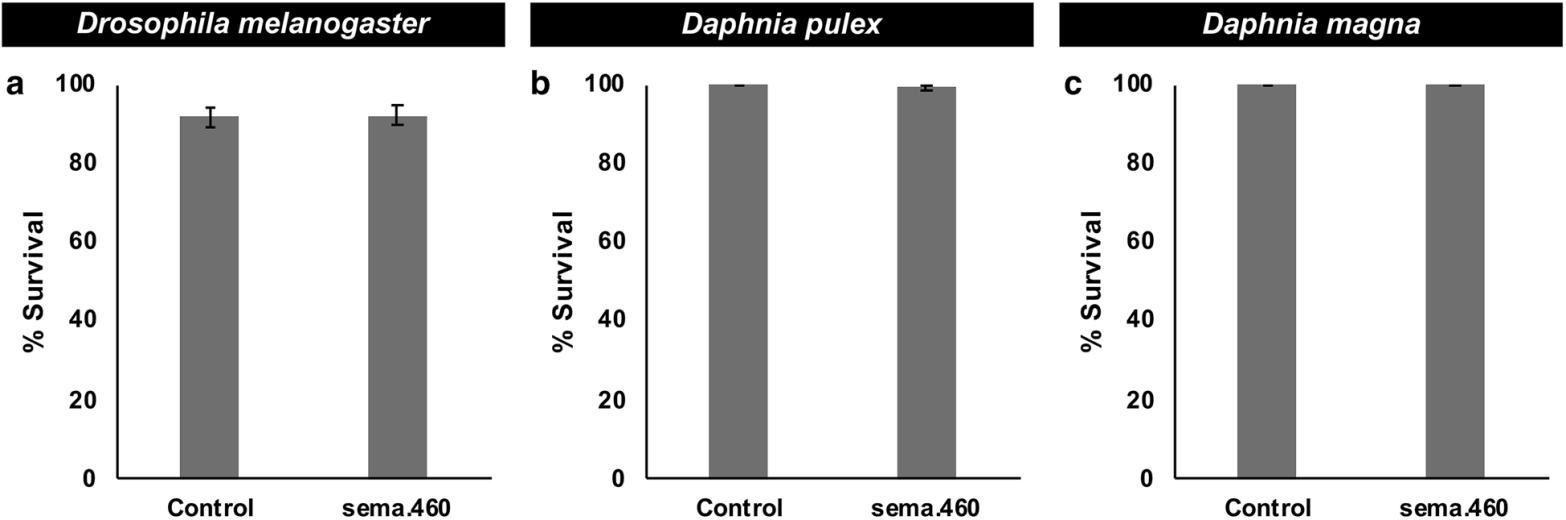
sema.460 is not toxic to three non-target arthropods. **a** Oral consumption of sema.460 by *D. melanogaster* larvae did not impact survival through adult emergence (a total of 20 larvae/treatment were assessed in two biological replicate experiments, each conducted with 10 larvae/treatment). **b** Consumption of sema.460 by *D. pulex* did not impact adult survival (30 animals/treatment were evaluated in three biological replicate experiments in which 10 animals/treatment were assessed over a 10-day trial period). **c** Likewise, sema.460 was not toxic to *D. magna* (30 animals/treatment were evaluated in three biological replicate experiments in which 10 animals/treatment were assessed for a 10-day trial period). Control groups in all of these assays were treated with yeast prepared from yeast expressing control shRNA. Survival data compiled from two (**a**) or three (**b**, **c**) replicate experiments and analyzed with the Fisher’s exact test indicated that there were no significant differences in the survival of sema.460- and control-treated organisms. Data shown represent mean percentages of survival

The sema.460 target site is conserved in multiple species of *Anopheles* mosquitoes, as well as *Ae. albopictus* and *Cx. quinquefasciatus*, but was not found to be conserved in the genomes of humans or other non-target organisms for which genome sequences are presently available, including arthropods other than mosquitoes (Additional file 2: Table S1). Based on the conservation of this target site in other mosquitoes, it was hypothesized that sema.460 would have broad-range mosquito larvicidal activity. As expected, significant larval mortality was observed in *Ae. albopictus* larvae that had consumed sema.460 tablets, with 93 ± 1% of larvae dying (Fig. 4a, *t*_(8)_ = 2.30, *P* = 2.26E^−12^ with respect to control-treated larvae). Similarly, larval consumption of dried inactivated yeast larvicide sema.460 tablets induced 90 ± 1% larval mortality in *An. gambiae* (Fig. 4b, *t*_(11)_ = 2.20, *P* = 1.93E^−17^ *versus* control treatment). Finally, sema.460 induced 90 ± 1% mortality in *Cx. quinquefasciatus* (Fig. 4c, *t*_(11)_ = 2.20, *P* = 1.76E^−16^ *versus* control treatment). These data support the hypothesis that sema.460 is a broad-range mosquito larvicide. Although sema.460 induces death in a variety of mosquito species, it has no larvicidal activity in *D. melanogaster*, a dipteran insect (Fig. 5a, *χ*^2^ = 1.598, *df* = 1, *P* = 0.21) in which the sema.460 target site was not identified (Additional file 2: Table S1). Likewise, *D. pulex* (Fig. 5b, *χ*^2^ = 0.4549, *df* = 1, *P* = 0.5) and *D. magna* (Fig. 5c, *χ*^2^ = 0, *df* = 1, *P* = 1), two distantly related aquatic arthropods that are often used in U.S. Environmental Protection Agency (EPA) toxicity assays [51], lack the sema.460 target site (Additional file 2: Table S1) and survived following consumption of sema.460.

## Discussion

### Yeast larvicide sema.460, a promising new tool for broad-based mosquito control

In this study, we provide detailed characterization of yeast larvicide sema.460, which may represent a new tool for broad-based mosquito control. Like other siRNAs that were previously designed to silence *sema1a* [23, 24], yeast interfering RNA larvicide sema.460 induces significant larval neural defects (Fig. 2) which, based on their close correlation with the onset of death (Fig. 1d), are presumably a primary cause of this death. However, sema.460, unlike the siRNAs that were previously used to assess the function of *sema1a* [23, 24], has a target site that is conserved in multiple mosquito species (Additional file 2: Table S1). *Aedes aegypti* and *An. gambiae*, which were previously shown to be susceptible to yeast interfering RNA larvicides targeting other larval lethal genes [25, 26], display high levels of mortality following treatment with sema.460 (Figs. 1, 4b). Additionally, the results of this study indicate that *Ae. albopictus* and *Cx. quinquefasciatus* larvae are also susceptible to yeast interfering RNA larvicides, with sema.460 inducing high levels of mortality in both species (Fig. 4a, c). While the sema.460 target site is conserved in multiple mosquito species, a lack of conservation of this site outside of mosquitoes (Additional file 2: Table S1), suggests that it could offer a new means of mosquito control that has little, if any, impacts on non-target organisms, including other arthropods. In support of this, sema.460 was not toxic to *D. melanogaster*, another dipteran insect, or *D. pulex* and *D. magna*, two distantly related aquatic arthropods that are recommended by the EPA for evaluation of the toxicity of substances to freshwater organisms [51] (Fig. 5).

As a member of a new class of larvicides, sema.460 could help combat resistance to existing insecticides, an ever-growing problem [10–16, 52]. Moreover, through our efforts to build an arsenal of interfering RNA larvicides ([25, 26], this study), we are working to combat resistance that could potentially develop due to a mutation in any one shRNA target site. A small percentage of sema.460-treated animals survive when the larvae are reared in a group of 20 animals (Fig. 1b, c). When these larvae are bred, the F1 progeny of the survivors are susceptible to sema.460 (Fig. 1f), suggesting that the F0 larvae which survived treatment were not resistant to sema.460 larvicide or RNAi in general (a trait which would have been inherited by their F1 progeny). Instead, it seems likely that survivors of sema.460 treatment may be eating other dead larvae in the container rather than yeast. In support of this, when 20 or more larvae are treated with sema.460 in a single container, dead larvae are rarely observed in these containers. However, when larvae were reared as individuals and treated with sema.460, 100% of larvae died (Fig. 1g), and the remains of these larvae were visible for several days, providing further evidence that F0 larvae of the LVP-IB12 strain used in these experiments do not harbor resistance to RNAi or the sema.460 larvicide. We are in the process of assessing in greater detail whether resistance to yeast interfering RNA larvicides can develop in *Ae. aegypti* over time.

### Potential benefits of a yeast delivery system for RNAi-based mosquito control

*Saccharomyces cerevisiae* may provide an excellent system for both cost-effective interfering RNA production and delivery of the RNA to mosquito larvae. As recently discussed [27], *S. cerevisiae*, which lacks both Dicer and Argonaute [53] and therefore a functional endogenous RNAi system, may have initially caused it to be overlooked as a system for producing interfering RNA molecules. However, this deficiency in RNAi machinery could potentially promote the accumulation of bioengineered interfering RNA in yeast, making it an excellent system for interfering RNA production. Similarly, studies in plants have indicated that dsRNAs can be more stably produced in chloroplasts, a cellular component which also lacks functional RNAi machinery [54]. Expression of dsRNA targeting potato beetles in the chloroplasts of potato plants, as opposed to the nuclear genome, led to accumulation of dsRNA at high levels and increased potato beetle mortality [54]. These results, which have sparked interest in use of the chloroplast genome to express dsRNAs targeting agricultural pests [54], may similarly apply to yeast and could explain why this system silences mosquito genes so effectively ([25, 26], this study).

As discussed in a recent review [27], genetic engineering of *S. cerevisiae* facilitates affordable synthesis of shRNA, which is produced as the yeast is cultivated. Yeast serves not only as an shRNA production system, but also as a strong mosquito larval odorant attractant [24] and a common source of larval nutrition in laboratory-bred mosquitoes, and so the production system can serve directly as the larval bait. Yeasts have been cultivated worldwide since ancient times, suggesting that yeast larvicides could be produced easily at many sites across the globe. Moreover, *S. cerevisiae*, a non-toxic product that is used frequently in beverage and food preparation and sold as a dietary supplement [27], may be more readily accepted than traditional chemical pesticides. Interestingly, our work has demonstrated that heat-inactivated yeast in which interfering RNA has been expressed kills larvae as effectively as live yeast [25, 26]. This is useful, particularly if there were to be interest in the use of these larvicides for treatment of drinking water, as there would be no further growth of the inactivated microbes in treated water. Although it is anticipated that yeast larvicide products will be safe, particularly with respect to conventional pesticides [55], it will of course be critical to perform toxicology tests on commercially-ready formulations, and these formulations will need to be reviewed by the appropriate regulatory bodies in the countries of intended use. The United States EPA recently approved an RNAi-based agricultural pesticide tool [17], and it is likely that additional registry applications will follow this one. However, the use of genetically modified yeast, even if it is dead, will need to be approved in each country of intended use. This will be a challenge, as some countries may lack a regulatory body equivalent to the EPA to evaluate this new technology.

The results of this investigation demonstrated that sema.460 induced high levels of larval mortality in both simulated-field and semi-field experiments (Fig. 3). These results suggest that it will be worthwhile to pursue further research and development in an effort to bring yeast larvicide technology to market. Critical areas for further development include optimization of RNA expression in *S. cerevisiae*, commercial-scaling of yeast larvicide production, and testing of commercially appropriate methods of yeast drying that preserve interfering RNA activity. It will also be important to identify encapsulating agents that will enhance yeast stability in various environmental conditions that might be encountered both prior to and during its use, and which may also serve to permit the controlled and extended release of yeast interfering RNA larvicides [27]. In preparation for the successful launch of field trials, it may also be useful to develop a variety of different formulations. For example, a variety of different Bti formulations, including tablets, granules, briquettes, powders, and flowable concentrates have been developed for control of mosquito larvae in various habitats [56]. Like Bti [56], it could also be helpful to vary the buoyancies of these formulations, which could facilitate the targeting of different species of disease vector mosquitoes that live in a wide variety of different habitats. The genetic tractability of *S. cerevisiae*, a genetic model organism, combined with the long and rich history of using yeast in food and pharmaceutical industries will undoubtedly help to advance development of this promising new technology for combating mosquitoes [27]. As formulation development, the piloting of scaled fermentation, and field-testing progress, it will also be critical to assess the relative cost of this technology in comparison to other larvicides.

### Beyond *Ae. aegypti*: Prospects for using yeast-based interfering RNA larvicides for control of other disease vector mosquitoes

The results of this investigation suggest that in addition to *Ae. aegypti*, yeast interfering RNA larvicides may also be used for the control of multiple disease vector mosquitoes (Fig. 4). While dried inactivated yeast pellets work well in laboratory assays, further work will be required to assess the most optimal formulations to be used for control of each species in the varied habitats in which it resides. The spatial distributions of *Ae. aegypti* and *Ae. albopictus*, both of which vector dengue, Zika, and chikungunya viruses, frequently overlap [57, 58]. The potential for controlling both species with a single interfering RNA larvicidal agent is advantageous, as the two species can lay eggs in the same container breeding sites [57]. Thus, ready-to-use inactivated yeast tablets, which were designed to facilitate the treatment of containers near consumer homes, might represent a means of controlling both species. Moreover, Hapairai et al. [25] demonstrated in laboratory trials that inactivated yeast tablets can attract gravid *Ae. aegypti* females to lay eggs in treated containers, and this may also be true for *Ae. albopictus* females. This feature is useful, as it may help to ensure that *Aedes* females, which are known to exhibit skip-oviposition behavior [59], will prefer to lay eggs in treated containers.

The results of this study also demonstrate that yeast interfering RNA larvicide technology may offer a new means of controlling *Culex* mosquitoes (Fig. 4c). The existing global disease burden of lymphatic filariasis [3], recent spread of West Nile virus across the continental United States, and reported resistance to existing larvicides [52] make the development of new larvicides for control of *Culex pipiens* complex mosquitoes a vital necessity. Larvicides for control of *Culex* mosquitoes are often used to treat stormwater catch basins, which are known to be important breeding sites for *Culex* mosquitoes in urban and suburban regions [60–62]. For targeting *Culex* mosquitoes, it may therefore be useful to develop formulations of yeast larvicides that are capable of persisting in catch basins. Nasci et al. [4], who recently evaluated the efficacy of a variety of different larvicides and formulations for treatment of stormwater catch basins functioning as *Culex* breeding sites, concluded that monthly re-treatments of these basins with granular larvicide formulations may be optimal. The authors speculate that tablets and briquettes may be prone to being buried in sediment or flushed out of the catch basin; they suggest that granular formulations may disperse more readily and be less prone to being buried or flushed out of the catch basins. Thus, for control of *Culex*, it may be useful to develop granular and buoyant formulations of yeast larvicides for optimized catch basin treatment. Such formulations may also be appropriate for treatment of large drums and barrels, typical water storage containers in the tropics that also function as some of the most productive *Aedes* breeding containers [50].

As discussed above, larviciding is an integral part of integrated *Aedes* [1] and *Culex* [2] mosquito control programmes. Additionally, in the first half of the 20th century, larval source management (LSM) was also a broadly applied and highly effective method for control of anopheline mosquitoes and malaria prevention [63]. However, IRS with dichlorodiphenyltrichloroethane (DDT) replaced many LSM programmes in the latter part of the 20th century [63]. IRS and ITNs continue to be the backbone for malaria vector control, but due to increasing problems with resistance to the insecticides used in these applications, there is renewed interest in expanding larval control programmes, which can reduce the number of mosquitoes that enter homes and the number of outdoor-biting mosquitoes for prevention of residual transmission [63]. The WHO determined that larviciding is cost-effective for malaria control in urban settings where vector breeding sites are few, fixed, and findable, and when it is used as a supplement to ITNs and IRS [5]. If larvicide programmes for control of *Anopheles* mosquitoes are to be sustained and eventually expanded, the identification of new, environmentally safe, cost-effective larvicides is critical. The results of this investigation, combined with our past findings [25–27], suggest that yeast-based larvicides may represent a promising new tool for control of *Anopheles* mosquitoes. To date, we have shown that these larvicides induce high levels of *An. gambiae* mortality, and it will be interesting to expand our laboratory analyses to additional *Anopheles* species, particularly given that the sema.460 target site is conserved in most available anopheline genome sequences (Additional file 2: Table S1). It will also be critical to expand formulation development so that optimal formulations are developed for each *Anopheles* species. Long-lasting FourStar™ briquette larvicides significantly reduced mean densities of both indoor- and outdoor-biting malaria vector mosquitoes in Western Kenya [64]. More recently, long-lasting LL3 and FourStar briquettes were found to significantly reduce immature densities of *An. gambiae* and *An. funestus*, with significant reductions observed for three months post-application. For habitats prone to overflow or water flow resulting from heavy rains, these briquettes were attached to poles with loose thin strings at the water margins [65]. It may be possible to develop similar formulations of yeast-based larvicides.

## Conclusions

The results of this study have demonstrated that sema.460, a yeast interfering RNA larvicide with a target site conserved in *Ae. aegypti*, *Ae. albopictus*, *Cx. quinquefasciatus*, as well as a variety of *Anopheles* spp. mosquitoes, functions as a broad-based mosquito insecticide (Figs. 1, 2, 4). The sema.460 target site is not found to be conserved in humans or in the available genomes of other non-target organisms, including insects other than mosquitoes (Additional file 2: Table S1). The activity of sema.460 was confirmed in laboratory trials that were designed to more closely simulate field conditions, as well as in outdoor semi-field trials (Fig. 3). This study, in conjunction with our past work [25–27], indicates that *S. cerevisiae* could serve as an excellent system for production of interfering RNA larvicides, as well as the delivery of these larvicides to a variety of different species of mosquito larvae. These yeast larvicides may offer a new biorational means of controlling a variety of disease vector mosquito species and combating insecticide resistance. Moreover, development of sema.460, a broad-based yeast interfering RNA mosquito larvicide, has furthered our ongoing efforts [25–27] to build an arsenal of interfering RNA pesticides that could be used to combat insecticide resistance that emerges from point mutations in the target site of any single insect target gene.

## Additional files

**Additional file 1.** Sequences of sema.460 and Control transgenes. The sequences of the sema.460 and control shRNA-encoding DNA cassettes used to generate pRS 426 plasmid clones are provided. Sequences of the sema.460 and control shRNA expression cassettes that were stably integrated at the *ura3* and *trp1* loci in the *S. cerevisiae* genome are also shown.

**Additional file 2: Table S1.** Assessment of sema.460 target site conservation. The 21 bp sequence targeted by sema.460 was used as a blastn query against all *Aedes*, *Anopheles* and *Culex* genomes in Vectorbase. Mosquito species bearing perfectly conserved target sequences are listed; the gene numbers (if known) or scaffold locations (s) corresponding to each match are indicated. The sema.460 target sequence was also used as an input for NCBI blastn searches conducted on the indicated taxonomic groups; corresponding taxonomic identification numbers (TaxIDs) are listed for each group. As of December 2018, searches against all sequences in the blast database did not uncover any perfect matches to the sema.460 target sequence outside of the indicated disease vector mosquito species.

## Data Availability

All data generated or analyzed during this study are included in this article.

## Abbreviations

Brp: Bruchpilot (Brp)
DDT: dichlorodiphenyltrichloroethane
CL: confidence limit
IRS: indoor residual spraying
ITN: insecticide-treated net
LSM: larval source management
LVP-IB12: Liverpool-IB12
RNAi: RNA interference
*sema1a*: *semaphorin-1a*
siRNA: small interfering RNAs
shRNA: short hairpin RNA
SEM: standard error of the mean
WHO: World Health Organization
