# Supplementary material for: Characterization of a broad-based mosquito yeast interfering RNA larvicide with a conserved target site in mosquito semaphorin-1a genes
Source: Parasit Vectors. 2019 May 22;12:256. doi: 10.1186/s13071-019-3504-x (PMC6532267; doi:10.1186/s13071-019-3504-x)
Supplement: Supplementary file 1 — Additional file 1. Sequences of sema.460 and Control transgenes. The sequences of the sema.460 and control shRNA-encoding DNA cassettes used to generate pRS 426 plasmid clones are provided. Sequences of the sema.460 and control shRNA expression cassettes that were stably integrated at the ura3 and trp1 loci in the S. cerevisiae genome are also shown. [file 13071_2019_3504_MOESM1_ESM.docx]

**Additional file 1**

**Sequence of sema.460 shRNA-encoding DNA Cassette Inserted in pRS 426 Plasmid:**

5’ATTATCGTCGCGGTGACGGATTTCAAGAGAATCCGTCACCGCGACGATAATTTTTTT3’

**Sequence of sema.460 shRNA Expression Cassette Integrated into *S. cerevisiae* Genome:**

5’ctcgagACGGATTAGAAGCCGCCGAGCGGGTGACAGCCCTCCGAAGGAAGACTCTCCTCCGTGCGTCCTCGTCTTCACCGGTCGCGTTCCTGAAACGCAGATGTGCCTCGCGCCGCACTGCTCCGAACAATAAAGATTCTACAATACTAGCTTTTATGGTTATGAAGAGGAAAAATTGGCAGTAACCTGGCCCCACAAACCTTCAAATGAACGAATCAAATTAACAACCATAGGATGATAATGCGATTAGTTTTTTAGCCTTATTTCTGGGGTAATTAATCAGCGAAGCGATGATTTTTGATCTATTAACAGATATATAAATGCAAAAACTGCATAACCACTTTAACTAATACTTTCAACATTTTCGGTTTGTATTACTTCTTATTCAAATGTAATAAAAGTATCAACAAAAAATTGTTAATATACCTCTATACTTTAACGTCAAGGAGAAAAAACCtctagaactagtggatccGATCCATTATCGTCGCGGTGACGGATTTCAAGAGAATCCGTCACCGCGACGATAATTTTTTTCctcgagtcatgtaattagttatgtcacgcttacattcacgccctccccccacatccgctctaaccgaaaaggaaggagttagacaacctgaagtctaggtccctatttatttttttatagttatgttagtattaagaacgttatttatatttcaaatttttcttttttttctgtacagacgcgtgtacgcatgtaacattatactgaaaaccttgcttgagaaggttttgggacgctcgaaggctttaatttgcggccggtacc3’

**Sequence of Control shRNA-encoding DNA Cassette Inserted in pRS 426 Plasmid**

5’GAAGAGCACTGATAGATGTTAGCGTTTCAAGAGAACGCTAACATCTATCAGTGCTCTTCTTTTTT3’

**Sequence of Control shRNA Expression Cassette Integrated into *S. cerevisiae* Genome**

5’ctcgagACGGATTAGAAGCCGCCGAGCGGGTGACAGCCCTCCGAAGGAAGACTCTCCTCCGTGCGTCCTCGTCTTCACCGGTCGCGTTCCTGAAACGCAGATGTGCCTCGCGCCGCACTGCTCCGAACAATAAAGATTCTACAATACTAGCTTTTATGGTTATGAAGAGGAAAAATTGGCAGTAACCTGGCCCCACAAACCTTCAAATGAACGAATCAAATTAACAACCATAGGATGATAATGCGATTAGTTTTTTAGCCTTATTTCTGGGGTAATTAATCAGCGAAGCGATGATTTTTGATCTATTAACAGATATATAAATGCAAAAACTGCATAACCACTTTAACTAATACTTTCAACATTTTCGGTTTGTATTACTTCTTATTCAAATGTAATAAAAGTATCAACAAAAAATTGTTAATATACCTCTATACTTTAACGTCAAGGAGAAAAAACCtctagaactagtggatcGAAGAGCACTGATAGATGTTAGCGTTTCAAGAGAACGCTAACATCTATCAGTGCTCTTCTTTTTTcgagtcatgtaattagttatgtcacgcttacattcacgccctccccccacatccgctctaaccgaaaaggaaggagttagacaacctgaagtctaggtccctatttatttttttatagttatgttagtattaagaacgttatttatatttcaaatttttcttttttttctgtacagacgcgtgtacgcatgtaacattatactgaaaaccttgcttgagaaggttttgggacgctcgaaggctttaatttgcggccggtacc3’
