# Supplementary material for: Characterization of a broad-based mosquito yeast interfering RNA larvicide with a conserved target site in mosquito semaphorin-1a genes
Source: Parasit Vectors. 2019 May 22;12:256. doi: 10.1186/s13071-019-3504-x (PMC6532267; doi:10.1186/s13071-019-3504-x)
Supplement: Supplementary file 2 — Additional file 2: Table S1. Assessment of sema.460 target site conservation. The 21 bp sequence targeted by sema.460 was used as a blastn query against all Aedes, Anopheles and Culex genomes in Vectorbase. Mosquito species bearing perfectly conserved target sequences are listed; the gene numbers (if known) or scaffold locations (s) corresponding to each match are indicated. The sema.460 target sequence was also used as an input for NCBI blastn searches conducted on the indicated taxonomic groups; corresponding taxonomic identification numbers (TaxIDs) are listed for each group. As of December 2018, searches against all sequences in the blast database did not uncover any perfect matches to the sema.460 target sequence outside of the indicated disease vector mosquito species. [file 13071_2019_3504_MOESM2_ESM.docx]

**Additional file 2: Table S1. Assessment of sema.460 target site conservation.** The 21 bp sequence targeted by sema.460 was used as a blastn query against all *Aedes, Anopheles,* and *Culex* genomes in Vectorbase. Mosquito species bearing perfectly conserved target sequences are listed; the gene numbers (if known) or scaffold locations (s) corresponding to each identical match are indicated. The sema.460 target sequence was also used as an input for NCBI blastn searches conducted on the indicated taxonomic groups; corresponding taxonomic identification numbers (TaxIDs) are listed for each group. As of December 2018, searches against all sequences in the blast database did not uncover any perfect matches to the sema.460 target sequence outside of the indicated disease vector mosquito species.

| **Mosquito Species or Taxonomic Group/Taxonomic ID** | **Identical**  **Match?** | **Match Location** |
| --- | --- | --- |
| *Aedes aegypti* | Yes | *AAEL019771* |
| *Aedes albopictus* | Yes | JXUM01S000300 (s), JXUM01S000372 (s) |
| *Anopheles atroparvus* | Yes | KI421889 (s) |
| *Anopheles arabiensis* | Yes | *AARA010942* |
| *Anoheles christyi* | Yes | *ACHR008382* |
| *Anopheles culicifacies* | Yes | AXCM01018274 (s) |
| *Anopheles darlingi* | Yes | scaffold_159 |
| *Anopheles dirus* | Yes | KB672957 (s) |
| *Anopheles epiroticus* | Yes | KB671536 (s) |
| *Anopheles farauti* | Yes | KI915063 (s) |
| *Anopheles funestes* | Yes | KB668692 (s) |
| *Anopheles gambiae* | Yes | *AGAP008656* |
| *Anopheles maculatus* | Yes | AXCL01008346 (s) |
| *Anopheles melas* | Yes | AXCO02012410 (s) |
| *Anopheles merus* | Yes | KI915170 (s) |
| *Anopheles minimus* | Yes | AMIN011338 (s) |
| *Anopheles quadriannulatus* | Yes | KB667866 (s) |
| *Anopheles sinensis* | Yes | AXCK02019776.1 (s) |
| *Anopheles stephensi* | Yes | scaffold_00097 (s) |
| *Culex quinquefasciatus* | Yes | supercont3.17 (s) |
| Amphibians/8292 | No | N/A |
| Birds/8782 | No | N/A |
| Fish/7898 | No | N/A |
| Fungi/4751 | No | N/A |
| Human/9606 | No | N/A |
| Insects/6960 (other than mosquitoes) | No | N/A |
| Mammals/40674 | No | N/A |
| Plants/3193 | No | N/A |
| Reptiles/8504 | No | N/A |
